# Three-Dimensional Ultrastructural Study of Oil and Astaxanthin Accumulation during Encystment in the Green Alga *Haematococcus pluvialis*

**DOI:** 10.1371/journal.pone.0053618

**Published:** 2013-01-11

**Authors:** Marina Wayama, Shuhei Ota, Hazuki Matsuura, Nobuhito Nango, Aiko Hirata, Shigeyuki Kawano

**Affiliations:** 1 Department of Integrated Biosciences, Graduate School of Frontier Sciences, University of Tokyo, Kashiwa, Chiba, Japan; 2 JST-CREST, Kashiwa, Chiba, Japan; 3 Ratoc System Engineering Co., Ltd., Tokyo, Japan; 4 Bioimaging Center, Graduate School of Frontier Sciences, University of Tokyo, Kashiwa, Chiba, Japan; Wuhan University, China

## Abstract

*Haematococcus pluvialis* is a freshwater species of green algae and is well known for its accumulation of the strong antioxidant astaxanthin, which is used in aquaculture, various pharmaceuticals, and cosmetics. High levels of astaxanthin are present in cysts, which rapidly accumulate when the environmental conditions become unfavorable for normal cell growth. It is not understood, however, how accumulation of high levels of astaxanthin, which is soluble in oil, becomes possible during encystment. Here, we performed ultrastructural 3D reconstruction based on over 350 serial sections per cell to visualize the dynamics of astaxanthin accumulation and subcellular changes during the encystment of *H. pluvialis*. This study showcases the marked changes in subcellular elements, such as chloroplast degeneration, in the transition from green coccoid cells to red cyst cells during encystment. In green coccoid cells, chloroplasts accounted for 41.7% of the total cell volume, whereas the relative volume of astaxanthin was very low (0.2%). In contrast, oil droplets containing astaxanthin predominated in cyst cells (52.2%), in which the total chloroplast volume was markedly decreased (9.7%). Volumetric observations also demonstrated that the relative volumes of the cell wall, starch grains, pyrenoids, mitochondria, the Golgi apparatus, and the nucleus in a cyst cell are smaller than those in green coccid cells. Our data indicated that chloroplasts are degraded, resulting in a net-like morphology, but do not completely disappear, even at the red cyst stage.

## Introduction

*Haematococcus pluvialis* Flotow is a freshwater unicellular biflagellate microalga belonging to the green algal class Chlorophyceae. It is well known to synthesize and accumulate high levels of the strong antioxidant astaxanthin (3,3′-dihydroxy-β,β-carotene-4,4′-dione) under stress conditions [1]. Various stress conditions; *e.g.*, nutrient deprivation [2], photon irradiance [3] [4], increased salinity [5] [6], high/low temperature [7], and combinations of stress [8] [9] [10], are known to accelerate astaxanthin synthesis and accumulation. Astaxanthin is closely related to other carotenoids, with which it shares many metabolic and physiological functions [11]. Due to its strong antioxidant activity and bright red coloration [12], it has been used as a pigment source in fish aquaculture (*e.g.*, salmon) and also has potential for pharmaceutical, cosmetic, food, and feed applications [11] [13] [14] [15].

*Haematococcus* astaxanthin is deposited in extra-plastidial oil bodies [16]. The *Haematococcus* lipid content and composition were analyzed and its potential as a resource for biodiesel feedstock was assessed [17]. It was also reported that the accumulation of oleic acid (C18:1), mainly in triacylglycerols (TAGs), was linearly correlated with the accumulation of astaxanthin monoesters under nitrogen starvation or high irradiance [18]. Although quantitative analyses of astaxanthin and lipids in *Haematococcus* have been performed, little is known about morphological changes and how much oil, including astaxanthin, accumulates in the transition from green coccoid cells to red cysts. One of the best ways to address the issue is direct visualization of entire cells by 3D transmission electron microscopy (3D-TEM).

Previous studies have addressed the astaxanthin accumulation pattern and carotenogenesis in *Haematococcus*. For example, carotenogenesis in living *H. pluvialis* cells was investigated by resonance-enhanced confocal Raman microscopy [19] [20]. An earlier TEM study by Lang [21] showed patterns of astaxanthin accumulation. This previous report also showed that gross differences in images were dependent on fixation (glutaraldehyde-KMnO_4_ vs. glutaraldehyde-OsO_4_) and emphasized the need for a variety of types of fixation upon which interpretation is based [21].

Here, we investigated oil and astaxanthin accumulation and subcellular structural changes during *H. pluvialis* encystment by 3D-TEM in conjunction with glutaraldehyde-KMnO_4_ and glutaraldehyde-OsO_4_ fixation. Using this technique, we were able to compare the relative volumes of each subcellular element between green coccoid and red cyst cells. The relative volume of astaxanthin in oil droplets increased dramatically from 0.2% in the green coccoid cells to 52.2% in cyst cells.

## Materials and Methods

### Culture conditions

An algal culture strain of *H. pluvialis* (K-0084) was obtained from the Scandinavian Culture Collection of Algae and Protozoa (SCCAP) at the University of Copenhagen. For observation of the life cycle, the cells were cultured in *Haematococcus* medium (Table S1). For TEM observations, the strain was cultured in TAP medium (without agar) [22] (http://mcc.nies.go.jp/02medium-e.html#tap). The cultures were grown at 20°C under 12-h light/12-h dark conditions (for green coccoid cells and intermediate cells) or continuous light (for cyst cells). The light intensity was set to ∼45 µmol photons m^−2^•s^−1^ using daylight fluorescent bulbs.

### Light and fluorescence microscopy

For visualization of nuclei in each stage, living cells were stained with SYBR Green I (final concentration 0.14%) (Molecular Probes, Eugene, OR), and were observed using a BX 51 fluorescence microscope (Olympus, Tokyo, Japan) equipped with differential interference contrast (DIC) optics. Images were captured with a DP70 CCD camera (Olympus, Tokyo, Japan). For visualization of oil droplets and astaxanthin, cells were stained with Nile Red (1 nM final concentration) (Polyscience, Inc., Warrington, PA), and were observed using a Leica DM6000B fluorescence microscope (Leica Microsystems GmbH, Wetzlar, Germany) equipped with DIC optics. The Nile Red signals, and astaxanthin and chlorophyll autofluorescence were detected with an L5 filter cube (excitation filter: 480/40 nm band pass (BP), suppression filter: 527/30 nm BP), an N3 filter cube (excitation filter: 546/12 nm BP, suppression filter: 600/40 nm BP), and a Y5 filter cube (excitation filter: 620/60 nm BP, suppression filter: 700/75 nm BP), respectively. Images were collected using a Leica DFC360 FX CCD camera (Leica Microsystems) as stacks with 0.79-µm increments in the *z*-axis, and the stacked images were deconvoluted with a Leica LAS AF v. 2.6.0 using the default settings (Leica Microsystems).

### Transmission electron microscopy (TEM)

We considered several fixation methods, and used two such methods in the present study. Glutaraldehyde (GA)-KMnO_4_ fixation was used for green coccid cells and GA-OsO_4_ fixation was used for intermediate (greenish-orange) cells and cyst cells. For GA-KMnO_4_ fixation, cells in 1.5 mL of medium were mixed with 1 mL of 2.5% GA and transferred into a glass vial. The mixture was microwaved for 30 s on ice and fixed for 6 h at 4°C. The cells were rinsed three times with distilled water. Subsequently, 2.5% KMnO_4_ was added and cells were fixed through incubation for 2 h. The cells were then rinsed six to seven times with distilled water. For OsO_4_ post-fixation, cells in 1.5 mL of medium were mixed with 1 mL of 2.5% GA and transferred into a glass vial. The mixture was microwaved for 30 s on ice and fixed for 6 h at 4°C. The cells were then rinsed three times with distilled water. Subsequently, 1% OsO_4_ was added and the cells were fixed overnight and rinsed three times with 0.05 M sodium cacodylate buffer (pH 7.8), once with 0.025 M sodium cacodylate buffer (pH 7.8), and finally three times with distilled water. After post-fixation, the cells were dehydrated using a graded ethanol series (30%, 50%, 70%, 90%, 95%, and 100%; 30 min each), and then incubated with ethanol∶acetone = 1∶1 and 100% acetone (30 min each). The dehydrated samples were infiltrated with increasing concentrations of Supper's resin in anhydrous acetone and finally with 100% Supper's resin (3 days in total). They were then polymerized at 50°C for 6 h and 60°C for 72 h. Ultrathin sections were cut on a Reichert Ultracut S ultramicrotome (Leica, Vienna, Austria) using a diamond knife. The sections were mounted on copper grids coated with polyvinyl formvar films, and stained in saturated aqueous uranyl acetate (30 min) and lead citrate (3 min) [23]. Sections were observed at 100 kV with an H-7650 transmission electron microscope (Hitachi High Technologies, Tokyo, Japan).

### 3D reconstruction

Digital TEM images were trimmed using Adobe Photoshop 6.0 (Adobe Systems Inc., San Jose, CA) and printed on A4 paper sheets. Contours of each subcellular element were traced manually using color marker pens (POSCA; Mitsubishi Pencil, Co., Ltd., Tokyo, Japan). The images were scanned and converted into digital images (JPG format). 3D images were subsequently reconstructed using TRI/3D SRF III software (Ratoc System Engineering, Co., Ltd., Tokyo, Japan). The absolute volumes (µm^3^) of each subcellular component were calculated using TRI/3D III software, and relative volumes were determined from the absolute volumes. The volumes of membrane structures, such as the plasma membrane, were calculated based on the thickness of the contours with the POSCA marked lines.

## Results

### Life cycle

**Figure 1:**
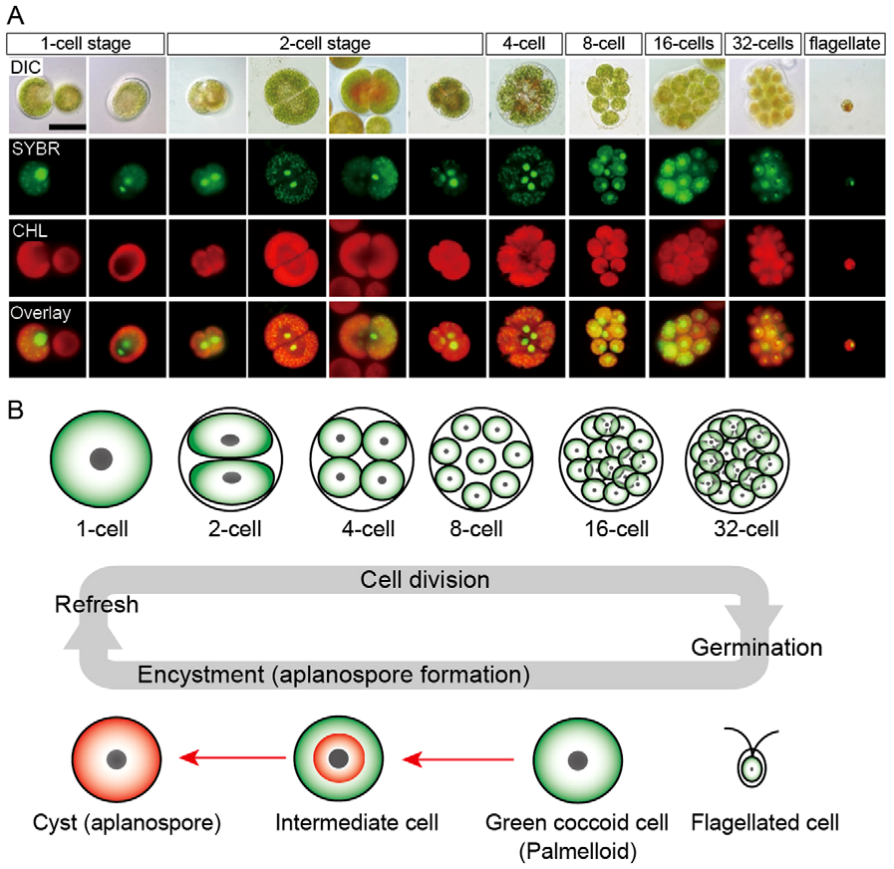
Life cycle of *H. pluvialis*. **A**. Fluorescence microscopy images, showing the 1- to 32-cell stages, and the flagellated stage. DIC: differential interference contrast image; SYBR: SYBR Green I-stained cells (green); CHL: chlorophyll autofluorescence (red); and Overlay: overlaid images of SYBR and CHL. **B**. Illustration of life cycle of *H. pluvialis*. Refresh: when old cultures are transplanted into fresh medium, coccoid cells undergo cell division to form flagellated cells within the mother cell wall. Germination: Flagellated cells settle and become coccoid cells. Continuous and/or strong light accelerate the accumulation of astaxanthin during encystment (red arrows).

Although gametogenesis in the *H. pluvialis* life cycle has been reported [24], little is known about its sexual reproduction. In the present study, we show the asexual life cycle of *Haematococcus* (Fig. 1A, B). When old cultures (2 weeks or more) were transplanted into fresh medium, the coccoid cells underwent cytokinesis and formed up to 32 daughter flagellated cells within the mother cell wall (Fig. 1A). About 36 h after transplantation, flagellated cells and non-hatched flagellated cells predominated in the culture. At 3–5 days after transplantation, most flagellated cells settled and became green coccoid cells (palmelloid). When green coccoid cells aged, they usually became greenish-orange as a result of astaxanthin accumulation. Some stress conditions, such as strong/continuous light, accelerated the process of encystment from the flagellated stage to the cyst (aplanospore) stage (Fig. 1B).

### Ultrastructure of vegetative cells

**Figure 2:**
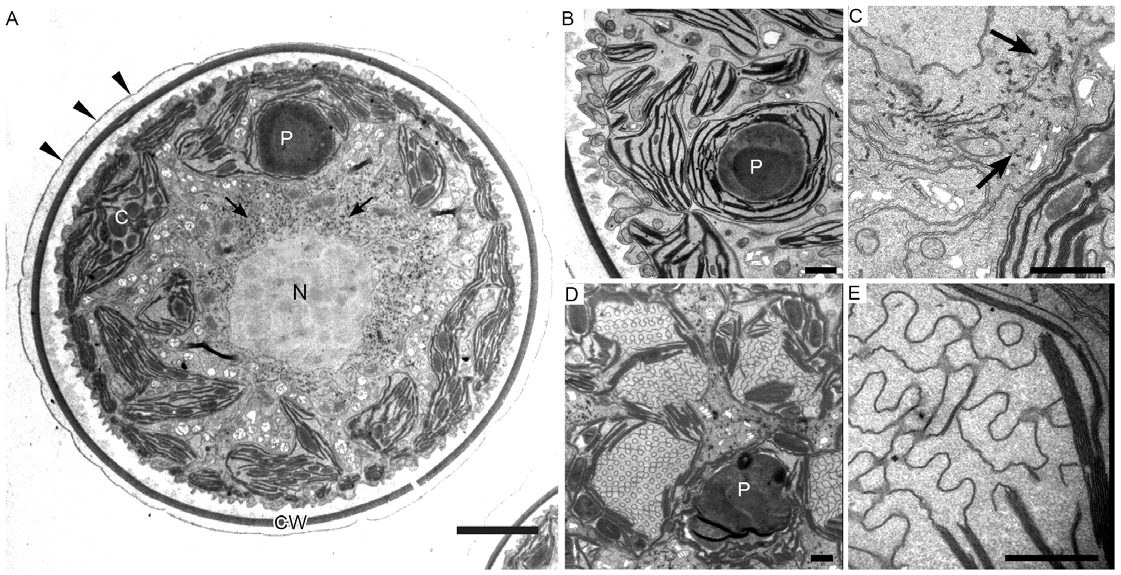
Transmission electron micrographs of green coccoid cells in *H. pluvialis*. **A**. General ultrastructure. The cell wall is surrounded by extracellular matrix (arrowheads). Arrows indicate astaxanthin granules. **B**. Chloroplast and pyrenoid. **C**. High-magnification view of astaxanthin granules (arrows). **D, E**. One-layer thylakoids with a regular arrangement. C, chloroplast; CW, cell wall; N, nucleus; P, pyrenoid. Scale bars in A and B–E: 5 µm and 1 µm, respectively.

Before 3D reconstruction analyses, we carefully observed ultrathin sections of various cell stages. Compared to GA-OsO_4_ fixation, GA-KMnO_4_ fixation enabled clear visualization of membrane structures, such as thylakoids (Fig. 2A, B). Few astaxanthin granules were present, which were located around the nucleus and were relatively electron-dense (Fig. 2A, C). Conspicuous pyrenoids with electron-dense matrix were observed in the stroma (Fig. 2A, B). A nucleus was located in the center of the cell, and highly developed chloroplasts were located at the periphery (Fig. 2A). A thick, electron-dense cell wall surrounded the cell, and two layers of extracellular matrix were observed near the cell wall (Fig. 2A). Sometimes, single-layer thylakoids were seen arranged loosely in the stroma (Fig. 2D, E).

### Ultrastructure of intermediate cells

**Figure 3:**
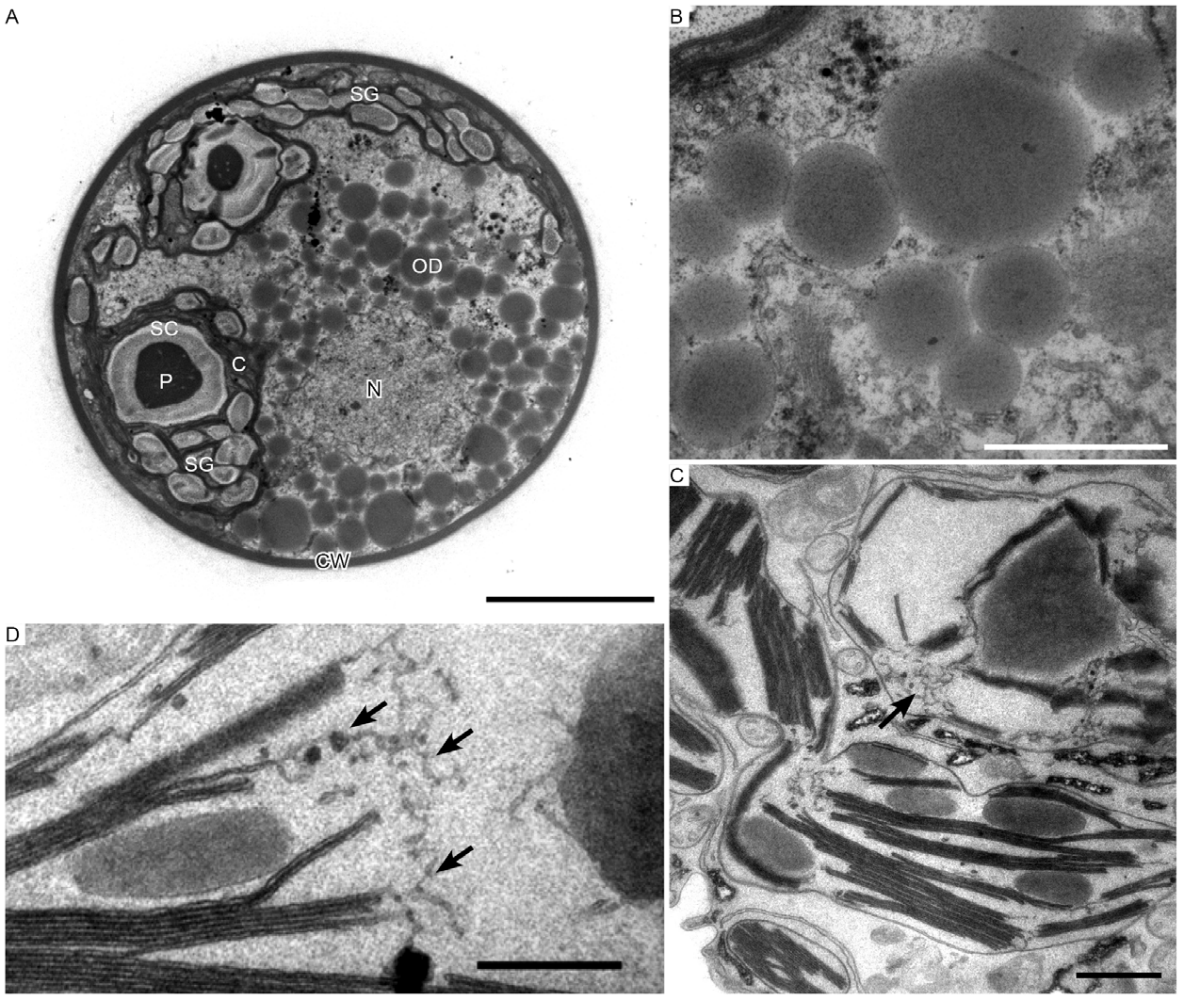
Transmission electron micrographs of intermediate *H. pluvialis* cells. **A**. General ultrastructure. **B**. High-magnification view of astaxanthin oil droplets. **C**. Partial degradation of thylakoids (arrow). **D**. High-magnification view of thylakoid degradation (arrows). C, chloroplast; CW, cell wall; N, nucleus; OD; oil droplet; P, pyrenoid; SC, starch capsule; SG, starch grain. Scale bars in A and B–D: 5 µm and 1 µm, respectively.

During encystment in *Haematococcus*, we often observed greenish-orange cells (with some astaxanthin accumulation) in middle-aged cultures (1–2 weeks). We designated these as intermediate cells. GA-OsO_4_ fixation successfully fixed oil droplets containing oil and astaxanthin. In the intermediate stage, conspicuous pyrenoids with electron-dense matrix were located in the stroma, and were surrounded by thick starch capsules. Many starch grains were located around the pyrenoids (Fig. 3A). Oil droplets containing astaxanthin were somewhat electron-dense and located around the nucleus. These droplets were rounded and of various sizes; there was no membrane-like structure around the oil bodies containing astaxanthin (Fig. 3B). At this stage, partial degradation of thylakoids was observed (Fig. 3C, D). At high magnification, thylakoids were observed to have become loose from edges of the thylakoid (Fig. 3D), suggesting partial thylakoid degradation.

### Ultrastructure of cyst cells

**Figure 4:**
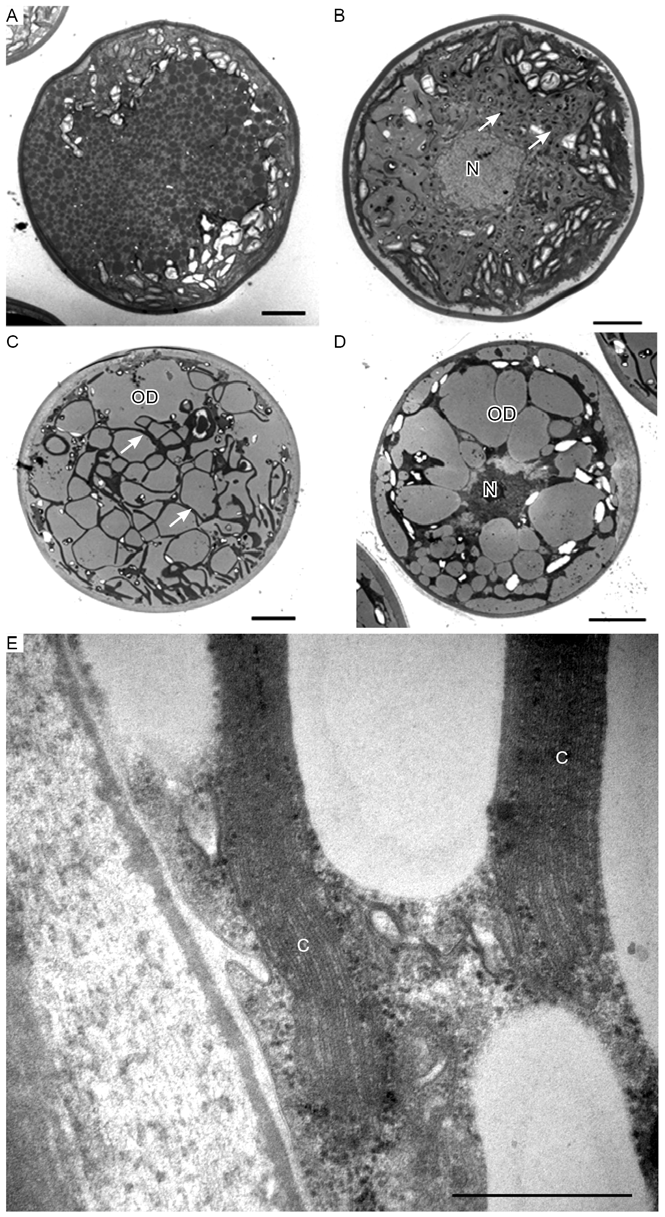
Transmission electron micrographs of *H. pluvialis* cyst cells. **A**. General ultrastructure of cyst cells, showing small granules that contain astaxanthin. **B**. General ultrastructure of a cyst cell, showing astaxanthin accumulation in oil droplets. **C**. General ultrastructure of a cyst cell, showing large oil droplets. Chloroplasts localize in the interspace between oil droplets (arrows). **D**. Some oil droplets are fused. **E**. High-magnification view of chloroplasts. C, chloroplast; N, nucleus; OD, oil droplet. Scale bars in A–D and E: 5 µm and 0.5 µm, respectively.

Strong and/or continuous light accelerated the accumulation of astaxanthin, resulting in the formation of cyst cells. For fixation of the cysts, we also used GA-OsO_4_ because it fixes droplets containing oil and astaxanthin as with the intermediate stage. The general ultrastructures of cells with high astaxanthin accumulation are illustrated in Fig. 4A–D, showing that oil droplets and astaxanthin accumulation patterns differ among cyst cells. For example, electron-dense astaxanthin granules in oil droplets (Fig. 4A, B) occurred in some cells. In other cells, relatively large oil droplets occurred throughout the cell (Fig. 4C, D), which seemed to contain astaxanthin. The oil droplets were less electron-dense and their sizes varied; droplets occasionally fused (Fig. 4D). Chloroplasts were highly degenerated and localized in the interspace between oil droplets, resulting in the chloroplasts having a net-like appearance (Fig. 4C). The chloroplasts contained compressed multilayered thylakoids, which were highly degraded during encystment (Fig. 4E).

### 3D reconstruction

**Figure 5:**
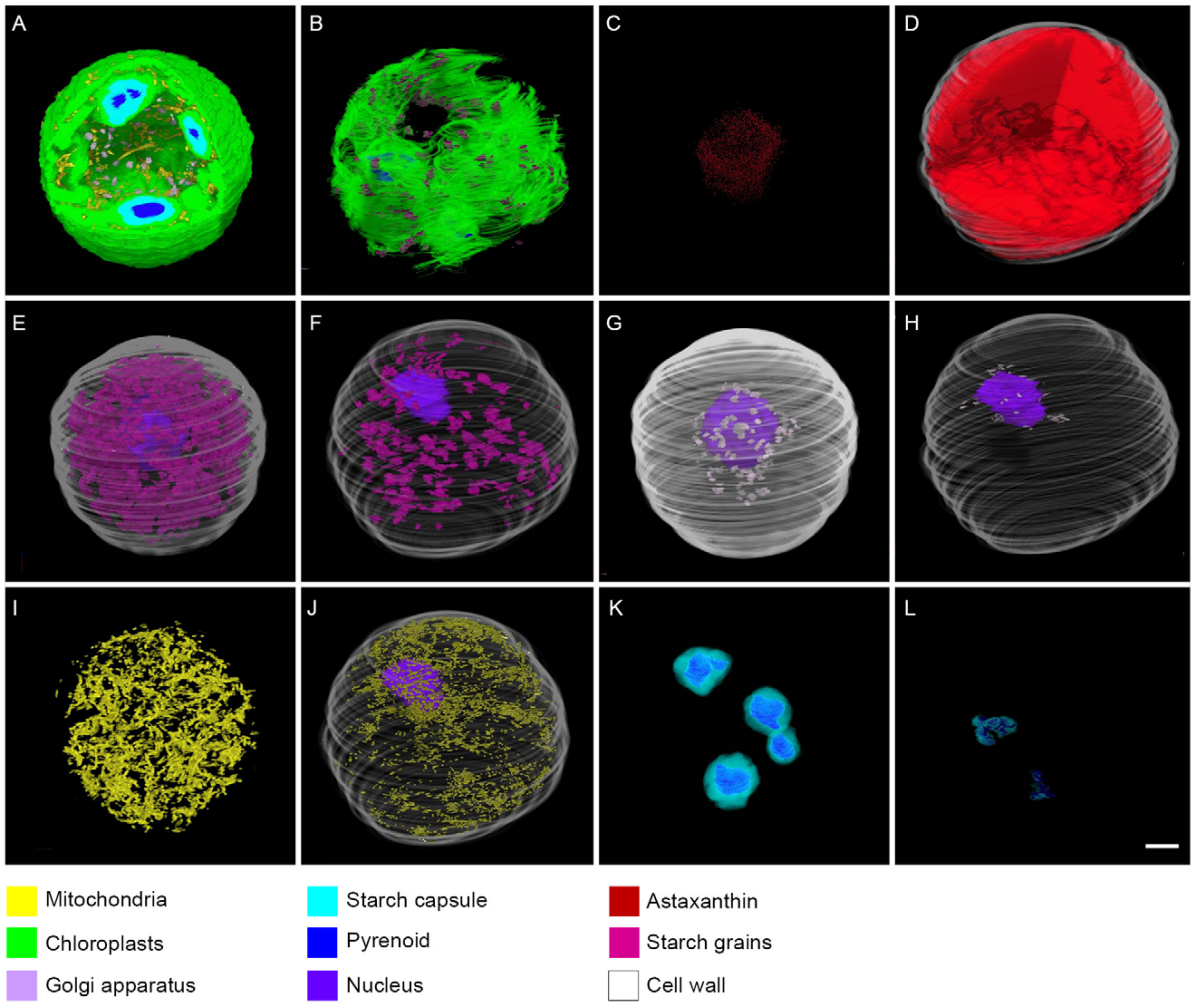
3D TEM images of subcellular components. **A**, **C**, **E**, **G**, **I**, and **K** represent a green coccoid cell; **B**, **D**, **F**, **H**, **J**, and **L** represent a cyst cell. **A** and **B**. 3D reconstruction of chloroplasts with pyrenoids, mitochondria, and/or starch grains. **C** and **D**. 3D reconstruction of astaxanthin distribution. **E** and **F**. 3D reconstruction of starch grains with the nucleus. **G** and **H**. 3D reconstruction of Golgi bodies with the nucleus. **I** and **J**. 3D reconstruction of mitochondria (with the nucleus in J). **K** and **L**. 3D reconstruction of pyrenoids and starch capsules. All subcellular components are denoted by different colors as indicated in the color legends. Scale bar in all images: 5 µm.

**Figure 6:**
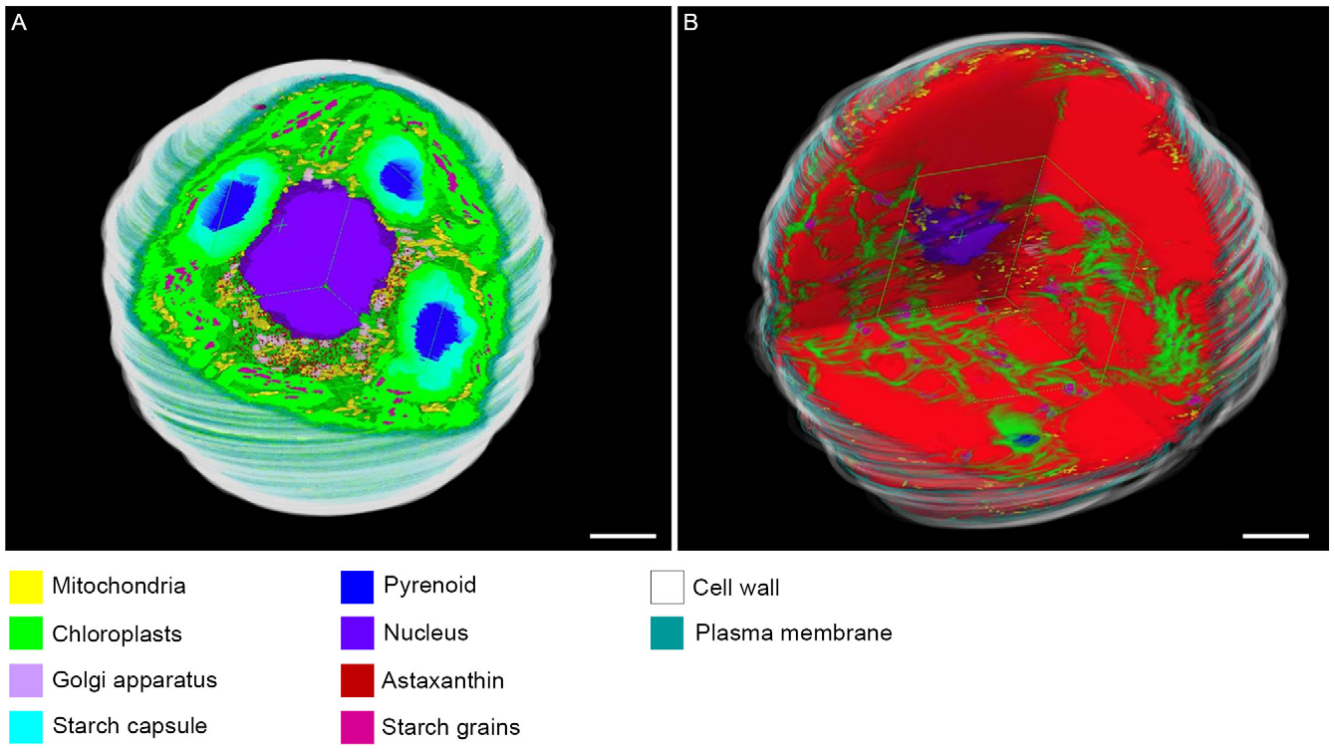
3D TEM images of whole cells. **A**. Cut-away image of a green coccoid cell. **B**. Cut-away image of a cyst cell. All subcellular components are denoted by different colors as indicated in the color legends. Scale bar: 5 µm. (See also Movies S1 and S2 for supporting information.)

To visualize whole cells, we employed the serial section method rather than electron tomography due to the large size of *Haematococcus* cells. Figure 5 shows a comparison of each subcellular component between green coccoid and red cyst cells based on 3D-TEM images. Chloroplasts were located in the entire green coccid cell periphery (Fig. 5A); in contrast, chloroplasts showed considerable degeneration in the cyst cells, resulting in a net-like appearance (Fig. 5B). Despite this degradation, chloroplasts did not disappear during the cyst stage (Fig. 5B). In the green coccoid cells, astaxanthin in oil droplets occurred in the center of the cell (Fig. 5C), whereas astaxanthin was distributed throughout the cyst cell (Fig. 5D). Many starch grains were observed in the chloroplast stroma in green coccid cells (Fig. 5B); however, the number of starch grains was markedly decreased in red cyst cells (Fig. 5E, F). Similarly, the number of Golgi bodies was decreased from the green coccoid to cyst cell (Fig. 5G, H). There was no significant difference in the number of mitochondria between green coccoid and cyst cells (Fig. 5I, J). The volume of pyrenoid matrix was also markedly smaller in red cyst cells (Fig. 5K, L). However, the pyrenoid matrix did not disappear completely during the cyst stage (Fig. 5L). Figure 6 shows cut-away images of subcellular elements of green coccoid and red cyst cells, in which all subcellular elements are displayed (see Supplemental movies S1 and S2 for cross-sectional views).

### Localization of oil droplets and astaxanthin in living cells

**Figure 7:**
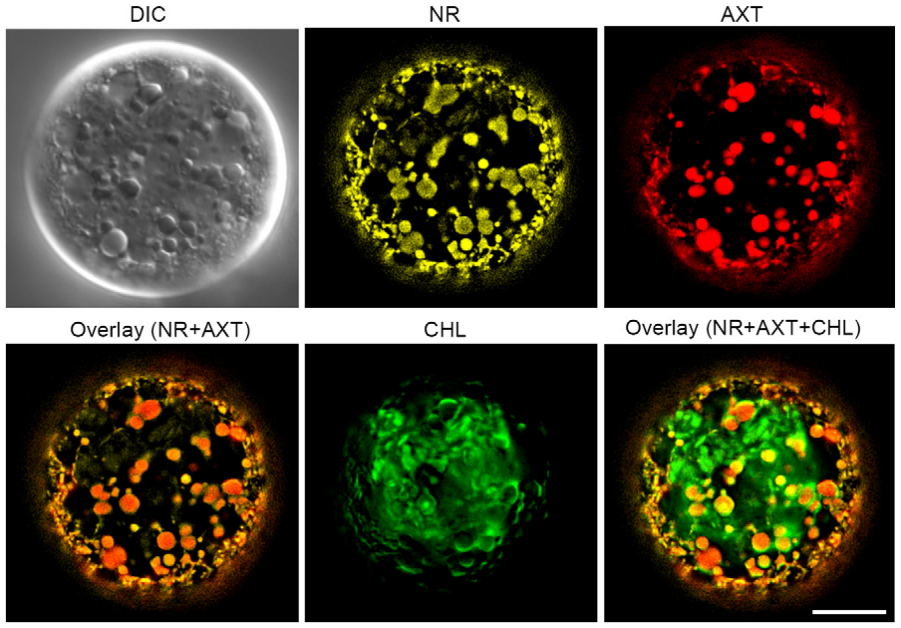
interference contrast (DIC) image and subcellular localization of lipids, astaxanthin, and chlorophyll in an astaxanthin-rich *H. pluvialis* cell. Nile Red (NR) signal, astaxanthin (AXT), and chlorophyll (CHL) autofluorescence are yellow, red, and green, respectively. Two overlaid images (NR+AXT and NR+AXT+CHL) are shown. Note that the Nile Red signals are colocalized with astaxanthin, shown in orange. Scale bar: 10 µm.

To validate the oil granules as astaxanthin-containing vesicles, fluorescence microscopy was used to observe the localization of Nile Red (a fluorescent lipophilic dye), astaxanthin autofluorescence and chlorophyll autofluorescence in an astaxanthin-rich cell. These observations indicated that astaxanthin autofluorescence were colocalized with Nile Red signals in a pattern that differed from that of chlorophyll autofluorescence (Fig. 7).

### Volumetric observation

**Figure 8:**
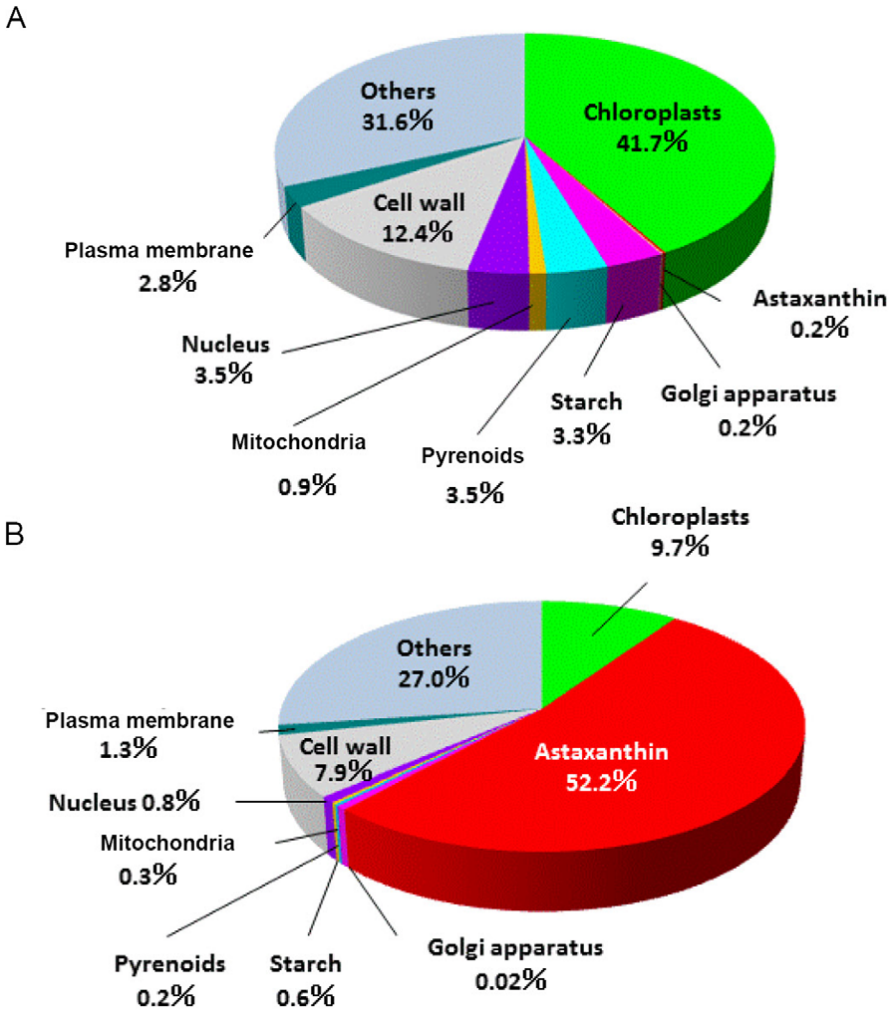
Relative volumes of *H. pluvialis* subcellular components. Subcellular components are indicated by colors in pie charts. **A**. Relative volumes of subcellular components in a green coccoid cell. **B**. Relative volumes of subcellular components in a cyst cell.

These 3D images revealed that drastic subcellular changes occurred in the transition between green coccoid cells and red cyst cells. Notably, the total volumes of chloroplasts and oil bodies containing astaxanthin changed markedly (Fig. 8). In the green coccoid cells, chloroplasts accounted for 41.7% of the total cell volume (absolute volume = 10018 µm^3^), whereas the total astaxanthin volume was very low (0.2%; absolute volume = 39 µm^3^). In contrast, oil droplets containing astaxanthin predominated in red cyst cells (52%; absolute volume = 14661 µm^3^), and the total volume of chloroplasts was drastically decreased in red cyst cells (9.7%; absolute volume = 2710 µm^3^) (Fig. 8). These results indicated that chloroplasts did not disappear in the red cyst stage, even with high chloroplast degeneration. The pyrenoid matrix volume decreased during encystment from 3.5% to 0.2%, but the pyrenoids did not disappear during the red cyst stage. Mitochondria appeared as particle-like structures that did not merge together. The present volumetric observations also demonstrated that the relative volumes of the cell wall, starch grains, pyrenoids, Golgi apparatus, and nucleus were smaller in cyst cells than in green coccid cells (Fig. 8).

## Discussion

We used over 350 serial sections to reconstruct 3D-TEM images of green coccoid and cyst cells to visualize the subcellular relationships and changes in *H. pluvialis*. A previous study by Collins [20] made detailed observations based on confocal Raman microscopy using living *Haematococcus* cells. Their findings suggested that astaxanthin is found within globular and punctate regions of the cytoplasmic space. Ultrastructural studies of β-carotene and astaxanthin accumulation in *H. pluvialis* showed that β-carotene was transported across the chloroplast membrane [25] and that astaxanthin occurs in extra-plastidial oil bodies [16]. The present fluorescence microscopic observations showing the colocalization of Nile Red with astaxanthin were consistent with previous findings.

Based on previous data [18], total levels of carotenoids (including astaxanthin) can reach ∼400 pg•cell^−1^ and total fatty acid levels can reach ∼3000 pg•cell^−1^ under conditions of nitrogen starvation, indicating that astaxanthin accounts for 13%–14% of oil bodies in *Haematococcus* under stress conditions. Although quantitative analyses of astaxanthin and lipids in *Haematococcus* have been well studied, little is known about subcellular changes during encystment. Our main interest in *H. pluvialis* is how and how much astaxanthin accumulates during encystment, and how subcellular structures change during encystment.

The present data showed that drastic changes in subcellular elements occur during the transition from the green coccoid stage to the red cyst stage during encystment. Notably, chloroplasts and oil droplets containing astaxanthin show large shifts among subcellular structures during encystment (Fig. 8). This result strongly suggests that astaxanthin accumulation and chloroplast degradation occur in the transition from the green coccoid stage to the red cyst stage. This result may be supported by a previous study, which showed that in cells subjected to strong light or nitrogen starvation, both astaxanthin and TAGs were rich in oleic acid, and that there was a direct positive correlation between oleic acid and esterified astaxanthin [18].

The present ultrastructural studies show that thylakoid degradation occurs in the intermediate stage and that chloroplasts are highly degenerated in the cyst stage, resulting in a marked reduction in total chloroplast volume (from 41.7% in green coccoids to 9.7% in red cysts, Fig. 8). Our findings also indicated that chloroplasts and pyrenoids do not disappear during the red cyst stage, despite the high rate of chloroplast degeneration. We speculate that the remaining chloroplasts play a role in rapid recovery when environmental conditions improve.

The results of fluorescence microscopy in the present study indicated that astaxanthin autofluorescence signals are colocalized with Nile Red signals, suggesting that astaxanthin is present in lipid droplets. This may be the case with fixed cells in TEM, and we consider that astaxanthin can be identified as oil droplets in transmission electron micrographs.

The results of volumetric analyses showed that oil bodies containing astaxanthin occurred throughout the cell, accounting for approximately half of the total cell volume. Considering that astaxanthin can account for up to 13%–14% of the total fatty acid level, as mentioned above, it is assumed that the most abundant components during the cyst stage are algal oils (roughly 44% of the total cell volume). Under conditions of stress (*e.g.*, continuous and/or strong light), the oil bodies contain TAGs as the major lipid class, using most of the oleic (18∶1) and palmitic (16∶0) acids [18]. Thus, *Haematococcus* may be a high TAG-producing alga and has potential for use in biofuel feedstock. In this study, volumetric observations were performed using only single cells. Further studies should include increased numbers of observations to facilitate statistical analysis of volumetric changes of subcellular components.

It is noteworthy that the cell wall structure and oil accumulation patterns changed. A thick, electron-dense cell wall surrounded the cell, and two layers of extracellular matrix were observed near the cell wall. However, the extracellular matrix was electron-transparent during the intermediate stage and was hardly visible during the cyst stage, suggesting that the extracellular matrix disintegrated as the coccoid cells aged, consistent with a previous observation [26]. During the cyst stage, oil droplets and astaxanthin accumulation patterns were different (Fig. 4). These differences might be related to the age of cells, and those containing large oil droplets (Fig. 4C, D) may represent the final form of the cyst stage.

Previous studies indicated accumulation of secondary carotenoids in the green alga *Chlorella zofingiensis* under conditions of stress [27] [28] [29] [30]. Ultrastructural analyses revealed that lipid bodies containing secondary carotenoids appeared around chloroplasts and accumulated in the cell periphery [27]. However, TEM studies revealed small astaxanthin granules immediately outside the nucleus in *Haematococcus* [21] (this study). *Haematococcus* and *Chlorella* show some common similarities with regard to stress responses; however, there are some differences between the two taxa.

In summary, based on our observations of TEM sections and 3D-TEM images, we concluded that: (i) green coccoids normally contain chloroplasts with developed thylakoids at the cell periphery; (ii) the chloroplasts begins to be degraded during the intermediate stage; and (iii) thylakoids degenerate as chloroplast degradation progresses and, as a consequence, chloroplasts exhibit a net-like appearance during the cyst stage. This suggests that the relative volumes of chloroplasts decrease during encystment. According to studies of ketocarotenoid biosynthesis in *Haematococcus*, astaxanthin accumulation occurs outside of chloroplasts [16]. Our findings also demonstrated that oil bodies containing astaxanthin are localized around the nucleus in green coccoids and during intermediate stages. Here, we report the first visualization of entire *Haematococcus* cells using the serial-section method. Visualization of multiple organelles simultaneously is not possible by light or fluorescence microscopy. The 3D serial-section method described here may be applicable to observation of the dynamics of ultrastructural changes, even in relatively large cells. Further studies should focus on intermediate stages to understand astaxanthin synthesis in the cytoplasm and degeneration of chloroplasts during encystment.

## Supporting Information

Movie S1 **3D reconstruction of a cell, showing cross-sectional images of a green coccoid cell (MPG).** Color legends used in the movie are the same as in Fig. 6.
(MPG)

Movie S2 **3D reconstruction of a cell, showing cross-sectional images of a cyst cell (MPG).** Color legends used in the movie are the same as in Fig. 6.
(MPG)

Table S1 **Chemical components of the** ***Haematococcus*** **medium.**
(DOC)
