# Supplementary material for: Three-Dimensional Ultrastructural Study of Oil and Astaxanthin Accumulation during Encystment in the Green Alga Haematococcus pluvialis
Source: PLoS One. 2013 Jan 11;8(1):e53618. doi: 10.1371/journal.pone.0053618 (PMC3543331; doi:10.1371/journal.pone.0053618)
Supplement: Table S1 — Chemical components of the Haematococcus medium. (DOC) [file pone.0053618.s003.doc]

**Table S1.** *Haematococcus* medium.

| Component | Stock (mol/L distilled water) | Quantity used |
| --- | --- | --- |
| NaNO_3_ | 1 M | 4.055 mL |
| CaCl_2_·2H_2_O | 1 M | 0.347 mL |
| Na_2_CO_3_ | 1 M | 0.189 mL |
| MgSO_4_·7H_2_O | 1 M | 0.304 mL |
| K_2_HPO_4_ | 1 M | 0.175 mL |
| EDTA 2Na | 0.5 M | 5.94 μL |
| Citric acid | 0.1 M | 0.312 mL |
| Co(NO_3_)_2_·6H_2_O | 0.1 M | 1.68 μL |
| Fe(III)NH_3_ citrate | 0.1 M | 38.17 μL |
| A5 metals (see below) | - | 1 mL |

Made up to 1 L with distilled water.

A5 metals.

| Component | Stock (mol/L distilled water) | Quantity used |
| --- | --- | --- |
| H_3_BO_3_ | 0.5 M | 4.7 mL |
| MnCl_2_·4H_2_O | 0.5 M | 0.91 mL |
| ZnSO_4_·7H_2_O | 0.5 M | 0.07 mL |
| Na_2_MoO_4_·2H_2_O | 0.5 M | 0.17 mL |
| CuSO_4_·5H_2_O | 0.5 M | 0.03 mL |

Make up to 50 mL with distilled water.
